# 2,3-Bis(bromo­meth­yl)-1,4-diphenyl­benzene

**DOI:** 10.1107/S1600536809050764

**Published:** 2009-12-04

**Authors:** Jonathan B. Briggs, Mikaël D. Jazdzyk, Glen P. Miller

**Affiliations:** a Department of Chemistry and Materials Science Program, University of New Hampshire, Durham, NH 03824-3598, USA

## Abstract

In the title compound, C_20_H_16_Br_2_, the terminal phenyl groups are twisted away from the central ring by approximately 55 and −125° (average of four dihedral angles each), respectively. The crystal structure is stabilized by a combination of inter­molecular and intra­molecular inter­actions including inter­molecular π–π stacking inter­actions [C atoms of closest contact = 3.423 (5) Å].

## Related literature

For the synthesis of terphenyls, see: Ames (1958 ▶). For the synthesis and applications of the title compound, see: Bredow *et al.* (1970 ▶); Geng *et al.* (2002 ▶); Martin & Segura (1999 ▶). For related structures, see: Baudour *et al.* (1986 ▶); Baker *et al.* (1993 ▶).
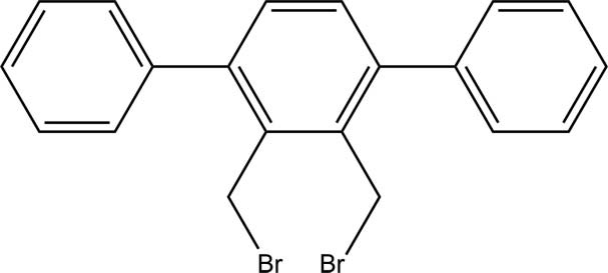


## Experimental

### 

#### Crystal data

C_20_H_16_Br_2_
*M* *_r_* = 416.15
Monoclinic, 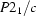

*a* = 8.8589 (10) Å
*b* = 11.5859 (13) Å
*c* = 16.655 (2) Å
β = 102.393 (4)°
*V* = 1669.6 (3) Å^3^
*Z* = 4
Mo *K*α radiation
μ = 4.85 mm^−1^
*T* = 296 K
0.50 × 0.50 × 0.05 mm

#### Data collection

Bruker SMART X2S diffractometer
Absorption correction: multi-scan (*SADABS*; Bruker, 2007 ▶) *T*_min_ = 0.195, *T*_max_ = 0.794
10674 measured reflections
2955 independent reflections
2395 reflections with *I* > 2σ(*I*)
*R*_int_ = 0.033

#### Refinement

*R*[*F* ^2^ > 2σ(*F* ^2^)] = 0.031
*wR*(*F* ^2^) = 0.117
*S* = 0.90
2955 reflections
199 parameters
H-atom parameters constrained
Δρ_max_ = 0.48 e Å^−3^
Δρ_min_ = −0.84 e Å^−3^

### 

Data collection: *GIS* (Bruker, 2007 ▶); cell refinement: *SAINT* (Bruker, 2007 ▶); data reduction: *SAINT*; program(s) used to solve structure: *SHELXS97* (Sheldrick, 2008 ▶); program(s) used to refine structure: *SHELXL97* (Sheldrick, 2008 ▶); molecular graphics: *SHELXTL* (Sheldrick, 2008 ▶); software used to prepare material for publication: *SHELXTL*.

## Supplementary Material

Crystal structure: contains datablocks I, global. DOI: 10.1107/S1600536809050764/fl2275sup1.cif

Structure factors: contains datablocks I. DOI: 10.1107/S1600536809050764/fl2275Isup2.hkl

Additional supplementary materials:  crystallographic information; 3D view; checkCIF report

## Figures and Tables

**Table 1:** Hydrogen-bond geometry (Å, °)

*D*—H⋯*A*	*D*—H	H⋯*A*	*D*⋯*A*	*D*—H⋯*A*
C12—H12⋯Br19	0.93	2.93	3.644 (4)	134
C20—H20*A*⋯Br19	0.97	2.80	3.563 (4)	136
C14—H14⋯Br20	0.93	2.96	3.704 (4)	139
C19—H19*A*⋯Br20	0.97	2.80	3.552 (4)	135
C19—H19*B*⋯Br20^i^	0.97	2.98	3.632 (4)	126

Symmetry code: (i) 
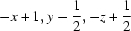
.

**Fig. 1.**
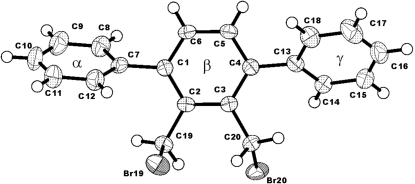
The molecular structure showing the crystallographic labeling scheme and displacement ellipsoids drawn at the 50% probability level. H atoms are shown as spheres of arbitrary radii.

**Fig. 2.**
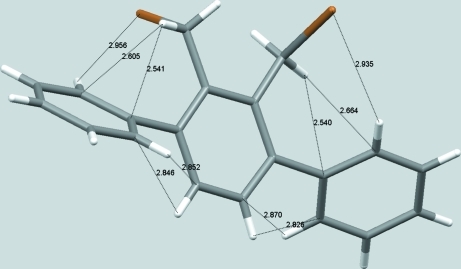
Perspective view of the title compound showing five nearly equivalent sets of intramolecular interactions involving the α and γ rings (see Comment section and Tables 1–2).

**Fig. 3.**
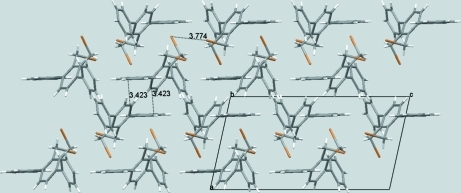
Perspective view of long range packing in the crystal structure including relatively weak intermolecular Br—Br and relatively strong intermolecular π-π stacking interactions.

**Fig. 4.**
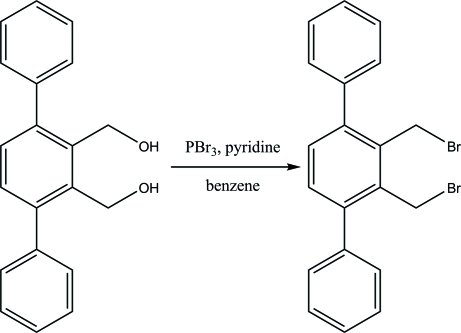
Synthesis of the title compound, 2,3-bis(bromomethyl)-1,4-diphenylbenzene.

## supplementary crystallographic information

### Comment

For a review on the synthesis of substituted terphenyls see Ames (1958). For the synthesis of the title compound, see Bredow *et al.* (1970). The title compound has been utilized as a reagent in the synthesis of spiro-configured terfluorenes (Geng *et al.*, 2002) and is a potentially useful precursor to an *o*-quinodimethane derivative (Martin & Segura, 1999). For related crystal structures, see Baudour *et al.* (1986); Baker *et al.* (1993).

We define the three rings of the terphenyl moiety as α, β and γ (Figure 1). Thus, terminal ring α contains C7 – C12, central ring β contains C1 – C6, and terminal ring γ contains C13 – C18. The rotations of ring α and ring γ relative to central ring β are approximately 55 ° and -125 °, respectively.

The rotations of ring α and ring γ are influenced by nearly equivalent sets of intramolecular C–H···π and C–H···Br interactions (Tables 1–2) as illustrated in Figure 2. Each γ ring also engages in a stabilizing, intermolecular π–π stacking interaction (C14···C16, 3.423 (5) Å) with another γ ring, as illustrated in Figure 3. The spacing between π–π stacking γ rings is nearly identical to the 3.435 Å interlayer spacing in graphite suggesting a relatively strong π–π stacking interaction. Likewise, interacting pairs of γ rings lie in near perfect parallel orientations with respect to each other (Figure 3).

There are 3 intermolecular and 4 intramolecular interactions involving Br atoms (Table 2). The intermolecular interactions consist of one significant Br–C interaction (Br19···C6, 3.470 (3) Å), one significant Br–H interaction (Br20···H19B, 2.9788 (5) Å), and one relatively weak Br–Br interaction (Br19···Br20, 3.7743 (7) Å). With regards to intramolecular interactions involving bromine, each bromine atom (*i.e.*, Br19 and Br20) interacts with one methylene hydrogen (Br19···H20A, 2.7993 (5) Å; Br20···H19A, 2.7965 (6) Å) and one aryl hydrogen (Br19···H12, 2.9348 (5) Å; Br20···H14, 2.9559 (6) Å). Both can be viewed as halogen variations of traditional H-bonding, the first set shorter and stronger presumably due to the greater acidity associated with a proton on a benzylic bromide as compared to an aryl proton. An *MM2* calculated structure for the title compound (not parameterized for Br···H interactions) indicates much longer Br···H distances (Br19,20···H19A,20 A, 3.14 Å; Br19,20···H12,14 3.79 Å) suggesting that Br···H-bonding in the crystal is both real and stabilizing.

Several intermolecular C–H···π interactions are also observed in the crystal structure (Table 1) but all have H···π distances greater than 3 Å and appear to be relatively weak.

### Experimental

The title compound was prepared *via* the published method (Bredow *et al.*, 1970) as illustrated in Figure 4. ^1^H NMR (400 MHz, CDCl_3_) δ 4.72 (s, 4H), 7.27 (s, 2H), 7.40–7.54 (m, 10H); ^13^C NMR (100 MHz, CDCl_3_) δ 29.2 (CH_2_), 128.0 (CH), 128.6 (CH), 129.2 (CH), 131.0 (CH), 135.1 (C), 140.4 (C), 143.7 (C). An X-ray grade crystal was grown from slow evaporation of a saturated chloroform solution.

### Crystal data

**Table:**

C_20_H_16_Br_2_	*F*(000) = 824
*M**_r_* = 416.15	*D*_x_ = 1.656 Mg m^−^^3^
Monoclinic, *P*2_1_/*c*	Mo *K*α radiation, λ = 0.71073 Å
Hall symbol: -P 2ybc	Cell parameters from 4281 reflections
*a* = 8.8589 (10) Å	θ = 2.4–24.7°
*b* = 11.5859 (13) Å	µ = 4.85 mm^−^^1^
*c* = 16.655 (2) Å	*T* = 296 K
β = 102.393 (4)°	Plate, colourless
*V* = 1669.6 (3) Å^3^	0.50 × 0.50 × 0.05 mm
*Z* = 4	

### Data collection

**Table:**

Bruker SMART X2S diffractometer	2955 independent reflections
Radiation source: micro-focus sealed tube	2395 reflections with *I* > 2σ(*I*)
doubly curved silicon crystal	*R*_int_ = 0.033
ω scans	θ_max_ = 25.1°, θ_min_ = 2.2°
Absorption correction: multi-scan (*SADABS*; Bruker, 2007)	*h* = −9→10
*T*_min_ = 0.195, *T*_max_ = 0.794	*k* = −12→13
10674 measured reflections	*l* = −19→18

### Refinement

**Table:**

Refinement on *F*^2^	Primary atom site location: structure-invariant direct methods
Least-squares matrix: full	Secondary atom site location: difference Fourier map
*R*[*F*^2^ > 2σ(*F*^2^)] = 0.031	Hydrogen site location: inferred from neighbouring sites
*wR*(*F*^2^) = 0.117	H-atom parameters constrained
*S* = 0.90	*w* = 1/[σ^2^(*F*_o_^2^) + (0.1*P*)^2^] where *P* = (*F*_o_^2^ + 2*F*_c_^2^)/3
2955 reflections	(Δ/σ)_max_ < 0.001
199 parameters	Δρ_max_ = 0.48 e Å^−^^3^
0 restraints	Δρ_min_ = −0.84 e Å^−^^3^

### Special details

**Table:**

Geometry. All e.s.d.'s (except the e.s.d. in the dihedral angle between two l.s. planes) are estimated using the full covariance matrix. The cell e.s.d.'s are taken into account individually in the estimation of e.s.d.'s in distances, angles and torsion angles; correlations between e.s.d.'s in cell parameters are only used when they are defined by crystal symmetry. An approximate (isotropic) treatment of cell e.s.d.'s is used for estimating e.s.d.'s involving l.s. planes.
Refinement. Refinement of *F*^2^ against ALL reflections. The weighted *R*-factor *wR* and goodness of fit *S* are based on *F*^2^, conventional *R*-factors *R* are based on *F*, with *F* set to zero for negative *F*^2^. The threshold expression of *F*^2^ > σ(*F*^2^) is used only for calculating *R*-factors(gt) *etc*. and is not relevant to the choice of reflections for refinement. *R*-factors based on *F*^2^ are statistically about twice as large as those based on *F*, and *R*- factors based on ALL data will be even larger.

### Fractional atomic coordinates and isotropic or equivalent isotropic displacement parameters (Å^2^)

**Table:**

	*x*	*y*	*z*	*U*_iso_*/*U*_eq_	
C1	0.7911 (4)	0.0211 (3)	0.11822 (18)	0.0261 (7)	
C2	0.6744 (3)	0.1014 (3)	0.12258 (18)	0.0251 (7)	
C3	0.6754 (3)	0.2110 (3)	0.08567 (18)	0.0251 (7)	
C4	0.7904 (4)	0.2404 (3)	0.04258 (19)	0.0269 (7)	
C5	0.9077 (4)	0.1602 (3)	0.04184 (19)	0.0296 (7)	
H5	0.9868	0.1787	0.0153	0.035*	
C6	0.9090 (4)	0.0545 (3)	0.0793 (2)	0.0292 (7)	
H6	0.9903	0.0039	0.0788	0.035*	
C7	0.7963 (4)	−0.0981 (3)	0.15312 (19)	0.0298 (7)	
C8	0.9294 (4)	−0.1362 (3)	0.2071 (2)	0.0408 (9)	
H8	1.0137	−0.0869	0.2219	0.049*	
C9	0.9371 (5)	−0.2470 (4)	0.2389 (3)	0.0522 (11)	
H9	1.0268	−0.2716	0.2747	0.063*	
C10	0.8145 (5)	−0.3204 (3)	0.2184 (3)	0.0533 (11)	
H10	0.8195	−0.3940	0.2410	0.064*	
C11	0.6828 (5)	−0.2845 (3)	0.1635 (3)	0.0530 (11)	
H11	0.5999	−0.3348	0.1483	0.064*	
C12	0.6736 (4)	−0.1744 (3)	0.1314 (2)	0.0419 (9)	
H12	0.5843	−0.1511	0.0947	0.050*	
C13	0.7925 (4)	0.3495 (3)	−0.0039 (2)	0.0312 (7)	
C14	0.7961 (4)	0.4578 (3)	0.0321 (2)	0.0380 (8)	
H14	0.7928	0.4636	0.0874	0.046*	
C15	0.8044 (5)	0.5578 (4)	−0.0128 (3)	0.0512 (11)	
H15	0.8063	0.6294	0.0125	0.061*	
C16	0.8098 (5)	0.5512 (4)	−0.0940 (3)	0.0532 (11)	
H16	0.8150	0.6181	−0.1241	0.064*	
C17	0.8075 (5)	0.4442 (4)	−0.1311 (3)	0.0562 (12)	
H17	0.8118	0.4392	−0.1863	0.067*	
C18	0.7989 (4)	0.3441 (4)	−0.0863 (2)	0.0429 (9)	
H18	0.7974	0.2726	−0.1119	0.051*	
C19	0.5527 (4)	0.0732 (3)	0.16939 (19)	0.0300 (7)	
H19A	0.5369	0.1395	0.2022	0.036*	
H19B	0.5883	0.0097	0.2066	0.036*	
C20	0.5528 (4)	0.2970 (3)	0.0926 (2)	0.0333 (8)	
H20A	0.4540	0.2581	0.0866	0.040*	
H20B	0.5441	0.3538	0.0491	0.040*	
Br19	0.35394 (4)	0.03039 (4)	0.09650 (2)	0.04810 (18)	
Br20	0.60479 (5)	0.37511 (4)	0.20073 (3)	0.05089 (18)	

### Atomic displacement parameters (Å^2^)

**Table:**

	*U*^11^	*U*^22^	*U*^33^	*U*^12^	*U*^13^	*U*^23^
C1	0.0308 (17)	0.0256 (18)	0.0219 (15)	−0.0022 (13)	0.0056 (13)	−0.0038 (12)
C2	0.0217 (15)	0.0319 (19)	0.0210 (14)	−0.0010 (13)	0.0028 (12)	−0.0052 (13)
C3	0.0240 (15)	0.0283 (18)	0.0218 (15)	0.0003 (13)	0.0027 (12)	−0.0034 (12)
C4	0.0296 (17)	0.0271 (18)	0.0241 (15)	−0.0016 (13)	0.0057 (12)	−0.0037 (13)
C5	0.0287 (16)	0.0316 (19)	0.0311 (17)	−0.0014 (14)	0.0126 (13)	−0.0036 (14)
C6	0.0271 (16)	0.0308 (18)	0.0319 (17)	0.0046 (13)	0.0110 (13)	−0.0033 (14)
C7	0.0372 (18)	0.0271 (18)	0.0269 (16)	0.0037 (14)	0.0111 (14)	−0.0048 (13)
C8	0.046 (2)	0.037 (2)	0.038 (2)	0.0043 (17)	0.0047 (16)	0.0014 (16)
C9	0.069 (3)	0.041 (2)	0.044 (2)	0.014 (2)	0.0068 (19)	0.0061 (18)
C10	0.081 (3)	0.027 (2)	0.056 (3)	0.010 (2)	0.025 (2)	0.0103 (19)
C11	0.064 (3)	0.029 (2)	0.070 (3)	−0.0084 (19)	0.023 (2)	−0.0034 (19)
C12	0.044 (2)	0.034 (2)	0.047 (2)	−0.0011 (17)	0.0089 (17)	0.0009 (17)
C13	0.0262 (16)	0.038 (2)	0.0300 (17)	0.0017 (14)	0.0061 (13)	0.0065 (14)
C14	0.040 (2)	0.033 (2)	0.044 (2)	0.0044 (15)	0.0148 (16)	0.0038 (16)
C15	0.046 (2)	0.036 (2)	0.076 (3)	0.0051 (18)	0.023 (2)	0.011 (2)
C16	0.043 (2)	0.054 (3)	0.064 (3)	0.0082 (19)	0.015 (2)	0.030 (2)
C17	0.051 (3)	0.080 (4)	0.036 (2)	−0.001 (2)	0.0060 (18)	0.025 (2)
C18	0.047 (2)	0.048 (2)	0.0323 (19)	−0.0016 (18)	0.0066 (16)	0.0047 (17)
C19	0.0283 (16)	0.0346 (19)	0.0274 (16)	−0.0030 (14)	0.0068 (13)	−0.0023 (14)
C20	0.0291 (17)	0.033 (2)	0.0388 (18)	0.0063 (14)	0.0089 (14)	0.0034 (15)
Br19	0.0284 (2)	0.0633 (3)	0.0510 (3)	−0.00986 (16)	0.00496 (17)	−0.00181 (18)
Br20	0.0586 (3)	0.0429 (3)	0.0588 (3)	0.00343 (18)	0.0298 (2)	−0.01598 (18)

### Geometric parameters (Å, °)

**Table:**

C1—C6	1.397 (5)	C11—C12	1.378 (5)
C1—C2	1.403 (5)	C11—H11	0.9300
C1—C7	1.496 (5)	C12—H12	0.9300
C2—C3	1.412 (4)	C13—C14	1.388 (5)
C2—C19	1.496 (5)	C13—C18	1.387 (5)
C3—C4	1.408 (4)	C14—C15	1.389 (5)
C3—C20	1.497 (4)	C14—H14	0.9300
C4—C5	1.396 (5)	C15—C16	1.366 (6)
C4—C13	1.485 (5)	C15—H15	0.9300
C5—C6	1.373 (5)	C16—C17	1.384 (6)
C5—H5	0.9300	C16—H16	0.9300
C6—H6	0.9300	C17—C18	1.390 (6)
C7—C8	1.392 (5)	C17—H17	0.9300
C7—C12	1.387 (5)	C18—H18	0.9300
C8—C9	1.385 (5)	C19—Br19	1.976 (3)
C8—H8	0.9300	C19—H19A	0.9700
C9—C10	1.364 (6)	C19—H19B	0.9700
C9—H9	0.9300	C20—Br20	1.979 (3)
C10—C11	1.382 (6)	C20—H20A	0.9700
C10—H10	0.9300	C20—H20B	0.9700
			
C6—C1—C2	118.2 (3)	C7—C12—C11	120.7 (4)
C6—C1—C7	118.2 (3)	C7—C12—H12	119.7
C2—C1—C7	123.6 (3)	C11—C12—H12	119.7
C1—C2—C3	120.0 (3)	C14—C13—C18	117.8 (3)
C1—C2—C19	120.2 (3)	C14—C13—C4	123.1 (3)
C3—C2—C19	119.7 (3)	C18—C13—C4	119.0 (3)
C2—C3—C4	120.8 (3)	C13—C14—C15	121.3 (4)
C2—C3—C20	119.5 (3)	C13—C14—H14	119.4
C4—C3—C20	119.7 (3)	C15—C14—H14	119.4
C3—C4—C5	117.6 (3)	C16—C15—C14	120.3 (4)
C3—C4—C13	124.3 (3)	C16—C15—H15	119.9
C5—C4—C13	118.0 (3)	C14—C15—H15	119.9
C6—C5—C4	121.6 (3)	C15—C16—C17	119.5 (4)
C6—C5—H5	119.2	C15—C16—H16	120.2
C4—C5—H5	119.2	C17—C16—H16	120.2
C5—C6—C1	121.6 (3)	C18—C17—C16	120.3 (4)
C5—C6—H6	119.2	C18—C17—H17	119.8
C1—C6—H6	119.2	C16—C17—H17	119.8
C8—C7—C12	118.3 (3)	C17—C18—C13	120.8 (4)
C8—C7—C1	119.6 (3)	C17—C18—H18	119.6
C12—C7—C1	122.0 (3)	C13—C18—H18	119.6
C7—C8—C9	120.4 (4)	C2—C19—Br19	112.4 (2)
C7—C8—H8	119.8	C2—C19—H19A	109.1
C9—C8—H8	119.8	Br19—C19—H19A	109.1
C10—C9—C8	120.7 (4)	C2—C19—H19B	109.1
C10—C9—H9	119.6	Br19—C19—H19B	109.1
C8—C9—H9	119.6	H19A—C19—H19B	107.8
C9—C10—C11	119.4 (4)	C3—C20—Br20	110.1 (2)
C9—C10—H10	120.3	C3—C20—H20A	109.6
C11—C10—H10	120.3	Br20—C20—H20A	109.6
C10—C11—C12	120.4 (4)	C3—C20—H20B	109.6
C10—C11—H11	119.8	Br20—C20—H20B	109.6
C12—C11—H11	119.8	H20A—C20—H20B	108.2
			
C6—C1—C2—C3	2.2 (4)	C7—C8—C9—C10	−0.3 (6)
C7—C1—C2—C3	−178.0 (3)	C8—C9—C10—C11	1.6 (6)
C6—C1—C2—C19	−175.1 (3)	C9—C10—C11—C12	−1.6 (6)
C7—C1—C2—C19	4.8 (4)	C8—C7—C12—C11	1.0 (5)
C1—C2—C3—C4	1.5 (4)	C1—C7—C12—C11	178.9 (3)
C19—C2—C3—C4	178.8 (3)	C10—C11—C12—C7	0.2 (6)
C1—C2—C3—C20	−178.4 (3)	C3—C4—C13—C14	58.9 (4)
C19—C2—C3—C20	−1.1 (4)	C5—C4—C13—C14	−122.9 (4)
C2—C3—C4—C5	−3.6 (4)	C3—C4—C13—C18	−124.3 (3)
C20—C3—C4—C5	176.3 (3)	C5—C4—C13—C18	53.9 (4)
C2—C3—C4—C13	174.6 (3)	C18—C13—C14—C15	0.5 (5)
C20—C3—C4—C13	−5.5 (5)	C4—C13—C14—C15	177.3 (3)
C3—C4—C5—C6	2.1 (4)	C13—C14—C15—C16	−0.2 (6)
C13—C4—C5—C6	−176.2 (3)	C14—C15—C16—C17	−0.2 (6)
C4—C5—C6—C1	1.6 (5)	C15—C16—C17—C18	0.4 (6)
C2—C1—C6—C5	−3.7 (5)	C16—C17—C18—C13	0.0 (6)
C7—C1—C6—C5	176.4 (3)	C14—C13—C18—C17	−0.4 (5)
C6—C1—C7—C8	53.1 (4)	C4—C13—C18—C17	−177.3 (3)
C2—C1—C7—C8	−126.8 (3)	C1—C2—C19—Br19	−103.0 (3)
C6—C1—C7—C12	−124.8 (4)	C3—C2—C19—Br19	79.7 (3)
C2—C1—C7—C12	55.3 (4)	C2—C3—C20—Br20	80.7 (3)
C12—C7—C8—C9	−1.0 (5)	C4—C3—C20—Br20	−99.2 (3)
C1—C7—C8—C9	−179.0 (3)		

### Hydrogen-bond geometry (Å, °)

**Table:**

*D*—H···*A*	*D*—H	H···*A*	*D*···*A*	*D*—H···*A*
C12—H12···Br19	0.93	2.93	3.644 (4)	134
C20—H20A···Br19	0.97	2.80	3.563 (4)	136
C14—H14···Br20	0.93	2.96	3.704 (4)	139
C19—H19A···Br20	0.97	2.80	3.552 (4)	135
C19—H19B···Br20^i^	0.97	2.98	3.632 (4)	126

Symmetry codes: (i) −*x*+1, *y*−1/2, −*z*+1/2.

### Table 2 C–H···π interaction geometry (Å, °)

For atom numbers, see Figure 1 in supplementary materials

**Table:**

C–H···π	C–H	H···π	C···π	C–H···π	Ring Involved
					
* C5-H5···C18	0.93	2.846 (4)	3.023 (5)	91.9 (2)	γ
* C6-H6···C8	0.93	2.826 (4)	3.046 (5)	94.7 (2)	α
* C8-H8···C6	0.93	2.870 (3)	3.046 (5)	91.9 (2)	α
* C18-H18···C5	0.93	2.852 (3)	3.023 (5)	91.5 (2)	γ
* C19-H19B···C7	0.97	2.540 (4)	2.987 (5)	108.0 (2)	α
* C19-H19B···C12	0.97	2.664 (4)	3.173 (5)	113.1 (2)	α
* C20-H20B···C13	0.97	2.541 (4)	2.988 (5)	108.1 (2)	γ
* C20-H20B···C14	0.97	2.605 (4)	3.172 (5)	117.6 (2)	γ
# C6-H6···C5	0.93	3.041 (4)	3.782 (5)	137.8 (2)	β
# C6-H6···C6	0.93	3.032 (4)	3.606 (5)	121.4 (2)	β
# C9-H9···C5	0.93	3.088 (3)	3.772 (6)	131.8 (3)	α
# C19-H19B···C17	0.97	3.018 (4)	3.600 (5)	119.8 (2)	γ

* indicates an intramolecular interaction; # indicates an intermolecular interaction
